# Supplementary material for: Directly imaging emergence of phase separation in peroxidized lipid membranes
Source: Commun Chem. 2023 Jan 17;6:15. doi: 10.1038/s42004-022-00809-x (PMC9845225; doi:10.1038/s42004-022-00809-x)
Supplement: Supplementary file 2 — Supplementary Information [file 42004_2022_809_MOESM2_ESM.pdf]

SUPPORTING INFORMATION

Directly imaging emergence of phase separation in peroxidized lipid membranes

Miguel Paez-Perez,[1] Aurimas Vyšniauskas,[1,2] Ismael López-Duarte,[1,3] Eulalie Lafarge,[4] Raquel Lopez-Rios De Castro,[5] Carlos M. Marques,[4,6] André P. Schroder,[4,7] Pierre Muller,[4] Christian D. Lorenz,[5] Nicholas J. Brooks, [1]* Marina K. Kuimova[1]*

[1]*MSRH, Department of Chemistry, Imperial College London, WoodLane, London, W12 0BZ, UK*
[2]*Center of Physical Sciences and Technology, Saulėtekio av. 3, Vilnius, Lithuania*
[3]*Departamento de Química en Ciencias Farmacéuticas, Universidad Complutense de Madrid, 28040 Madrid, Spain*
[4]*Institut Charles Sadron, CNRS and University of Strasbourg, 23 rue du Loess, F-67034 Strasbourg Cedex 2, France*
[5]*Department of Physics, King's College London, London WC2R 2LS, United Kingdom*
[6]*University of Lyon, ENS-Lyon, CNRS UMR 5182, Chem. Lab., 69342 Lyon, France*
[7]*University of Lyon, CNRS, INSA Lyon, LaMCoS, UMR5259, 69621 Villeurbanne, France*

Table of contents:

1. Supplementary Methods 2
2. Supplementary Figures 6
3. Supplementary References 16

1. Supplementary Methods

NMR characterization of POPC-OOH:

All Nuclear Magnetic Resonance (NMR) spectra were recorded on a Bruker Avance 400 spectrometer equipped with an Ultrashield magnet and a 5 mm BBFO probe. Chemical shifts (δ) are reported in parts per million (ppm) calibrated against Methanol-d4 residual signal (3.31 ppm). 1H-NMR spectra were recorded at 400 MHz.

Fluorescence Recovery After Photobleaching (FRAP) analysis:

Pure POPC and POPC-OOH GUVs doped with 0.1%mol NBD-PE lipid were electroformed using the protocol described in the main text and diluted by a factor of 15 (v/v) in 420mM glucose before imaging. Confocal imaging was performed using a Leica SP8 confocal module equipped with the FRAP module. Images were taken at a 256x256 pixel resolution and 1.54Hz frequency (650 ms/frame). Bleaching was performed for 2.6s (4 frames) and fluorescence recovery was recorded for 90s (139 frames).

In order to extract the FRAP curves and estimate the diffusion coefficient, the images were analyzed using a custom-written MATLAB script in the following way:

1. User was prompted to mark the vesicle centre and approximate inner/outer boundaries on the projection corresponding to the pre-bleached frames.
2. For the *k* frame, an angular scan was performed to find the vesicle edge, and the centre was set by fitting the detected boundary to the circumference equation.
3. For the frame *k+1*, an angular scan is performed using the conditions defined at (2). This allowed to track the vesicle position at each frame, thus overcoming potential artifacts arising from the GUV drifting and/or coming out of focus.
4. For each angular scan, an intensity vs angle trace is obtained. For the 1st post-bleaching frame, the bleached region (bROI) was defined by the centre ± s.d. of a gaussian fit over the intensity drop region. Simultaneously, an equivalent control ROI (cROI) was defined opposite to the bleached region, to account for overall photobleaching and/or finite lipid reservoir artifacts.
5. Steps 2-4 were repeated for each frame *i* to obtain the intensity traces I_{bROI} and I_{cROI}. The corrected intensity was then calculated as:

$$I_{corrected} = \frac{I_{bROI}}{I_{cROI}} \qquad \text{(s1)}$$

6. The corrected FRAP trace was then fitted to the model described by:

$$I(t) = (I_r - I_p)(1 - \exp\left(-\frac{t}{\tau_{FRAP}}\right) + I_p \qquad \text{(s2)}$$

where I(t) is the time-dependent corrected fluorescence intensity after recovery, I_r is the end-point fluorescent intensity and I_p is the fluorescent intensity immediately after bleaching.

7. Finally, the diffusion coefficient was approximated as:[1]

$$D = 0.224w^2/\tau_{1/2} \qquad \text{(s2)}$$

where $\tau_{1/2} = \tau_{FRAP}\log(2)$ and w is the radius of the bleached region, which we considered to be half the perimeter of the bleached GUV area. We note this definition is not strictly accurate, and that GUVs may not be considered an infinite source of unbleached molecules (as is required for FRAP idealization), yet we deemed this approach to be sufficient for comparative purposes.

Estimation of the diffusion coefficient from **BC10** lifetime:

If we assume BC10 behaves as a membrane-embedded object of radius a (~6.2Å, according to ref[2]), its diffusion coefficient could be estimated from viscosity values by using the Saffman-Delbrük equation:[2,3]

$$\boldsymbol{D_{SD}} = \frac{\boldsymbol{k_B T}}{\boldsymbol{4\pi\eta_m h}}\left[\ln\left(\frac{\boldsymbol{2L_{SD}}}{\boldsymbol{a}}\right) - \boldsymbol{\gamma}\right] \tag{s1}$$

where k_B refers to Boltzmann's constant, T is the temperature (295 K), η_m is the membrane viscosity (which was estimated from the **BC10** lifetime), *h* is the membrane's thickness (estimated from molecular dynamics simulations, Fig. 6 of the main text), γ is the Euler-Mascheroni constant (~0.577) and L_{SD} stands for the Saffman-Delbrük length, which, considering the viscosity of the surrounding fluid η_f (~0.89 cP), can be expressed as:

$$L_{SD} = \frac{h}{2}\frac{\eta_m}{\eta_f} \tag{s2}$$

<u>Laurdan generalized polarization and spectral decomposition analysis:</u>
Laurdan's GP was calculated as:

$$GP = \frac{I_{435\pm2} - I_{490\pm2}}{I_{435\pm2} + I_{490\pm2}} \tag{s3}$$

Spectral decomposition was done following the strategy suggested by Watanabe et al.[4], Laurdan's emission spectra was decomposed into three lognormal distributions according to Eq. s4**.**

$$I(x) = \sum_{i=1}^{3} a_i \exp\left[-\frac{1}{2}\left(\frac{ln\left(\frac{x}{c_i}\right)}{w_i}\right)^2\right] \tag{s4}$$

where *I(x)* is the fluorescence intensity at wavelength *x*, α_i is the pre-exponential amplitude factor, c_i is the centre of the log-normal curve and w_i corresponds to the curve's width. While the results were sensitive to the starting conditions, the distribution of results for each Laurdan population remained distinct. Fig. S3 shows the results of fitting with different starting conditions (>100 runs), randomly varying the starting point at each run. Fig. S3d-f shows the variations of c_i with increasing temperature, while Fig. S3g-i displays the variation of the relative changes of integrated areas under curves in Fig. S3d-f.

<u>Analysis of intra-vesicle viscosity heterogeneity:</u>
Intra-vesicle lifetime heterogeneity, an indicator for lipid clustering, resulting in regions of distinct viscosity, was quantified by using Shannon's entropy:

$$\mathcal{H}_{Shannon} = -\sum_{i=1}^{n} P(x_i)\log_{10} P(x_i) \tag{s5}$$

where $P(x_i)$ is the probability of observing a viscosity x_i within a single vesicle, and *n* is the number of existing viscosity values within the membrane. For a completely homogeneous membrane, $P(x_i) = 1$ for a single value of *i*, and therefore $\mathcal{H}_{Shannon} = 0$. On the other hand, for a GUV showing maximum heterogeneity $P(x_i) = 1/n$ and thus $\mathcal{H}_{Shannon} = \log_{10} n$. This metric was previously used to quantify lipid mixing in simulated membrane systems.[5,6]
All experiments were performed at 22±1°C. For GUV heating experiments, a Peltier element was used (heating rate 10°C/min) and vesicles were left to equilibrate for 5 minutes at each temperature point before imaging.

<u>Viscosity-lifetime transformation:</u>
Sample viscosity for **BC10** stained membranes was calculated from the calibration by *Hosni et al.*[7] while **BC6++** longest lifetime was transformed into viscosity following the calibration *by López-Duarte et al.*[8]

$$\mathbf{BC10}\text{:}\ \log_{10}\tau = 0.4569\log_{10}\eta - 0.75614 \tag{s6}$$

$$\mathbf{BC6}^{++}\text{:}\ \ln\tau = 0.5\ln\eta - 1.9422 \tag{s7}$$

X-ray diffraction experiments:

The diffraction profiles were obtained from the radial integration of the 2D SAXS and WAXS patterns, and peak position and width was determined by fitting them to pseudo-Voigt functions using a custom-built MATLAB® script. The area per lipid was approximated as suggested by *Mills et al.*[9] from the *q* position of the WAXS peak as:

$$\frac{A_L}{2} \approx 1.32\left(\frac{9\pi}{4q}\right)^2 \quad \text{(s8)}$$

Experimental error was quantified by performing triplicate measurements on pure POPC samples. The coefficient of variation for this control was then used to estimate the standard deviation of the measured parameters for samples containing the peroxidized lipid.

Flickering spectroscopy:

GUVs were diluted 20-fold in a 420mM glucose solution and added to a BSA-coated observation slide for imaging. The slight osmotic imbalance promoted vesicle deflation and membrane fluctuations, needed to calculate the membrane's bending modulus κ_b. High-speed (1KHz) phase-contrast video recordings of debris free, fluctuating vesicles were acquired for ~40s.

These videos were then analyzed with a custom-built LabView® script to extract κ_b. Briefly, 360 radial slides were taken, and for each of them the vesicle edge was determined with subpixel resolution, as the maximum of the 1st derivative of the image intensity across the radial slice. These points were then used to calculate the vesicle's centre, so that the change in radius was extracted as a function of the angular position for each frame. The power spectra of the Δr-θ traces were obtained by FFT. From the relation between the fluctuation modes and their amplitudes:

$$< h(q_x, y=0)^2 > = \frac{k_B T}{4\pi R}\frac{1}{\sigma}\left[\frac{1}{q_x} - \frac{1}{\sqrt{\frac{\sigma}{\kappa_b}+q_x^2}}\right] \quad \text{(s9)}$$

Where k_B is the Boltzmann's constant, T is temperature, R is the vesicle's radius, q_x is the fluctuation mode and σ represents membrane tension. Then, it is possible to fit the power spectrum to extract the membrane's bending rigidity and tension. Modes 5-15 were used for fitting the power spectrum. Fluctuation analysis suggest a decrease in bending rigidity for GUVs containing increasing amounts of POPC-OOH (Fig. S5).

μ-Differential scanning calorimetry (μ-DSC):

POPC and POPC-OOH in MeOD (10 mg/mL) were mixed at a relative fraction to obtain the desired hydroperoxydation degree. The relative fraction of each mixture was precisely determined from NMR before the experiments were carried out. Five hydroperoxidation fractions were produced, ranging from 0 to 20%. 250 μL of a 10 mg/mL lipid solution in MeOD was dried under reduced pressure, and after, 250 μL of milli-Q water was added to the precisely weighed dried lipid film. The obtained solution was vortexed for 1 min and sonicated for 15 min in an ultrasound bath to form Giant Multilamellar Vesicles (GMVs) of various sizes and degree of lamellarity. Differential Scanning Calorimetry (DSC) was performed using a micro-DSC (MC DSC, TA instruments) calibrated with pure sapphire. All calorimetric scans and standardizations were carried out with a first set of cooling/heating (C/H) cycles ranging from -20°C to 20°C, followed by a second set of cycles ranging from -12°C to 20°C. Each C/H cycle was performed at three different temperature rates: 1°C/min, 0.5°C/min and 0.25°C/min as detailed in Fig.S6. Since the main phase transition temperature T_m of POPC is negative, problems due to freezing of water during DSC scans were expected. In our device, freezing occurred randomly between -12 and -18°C. Due to this lower limit, some lipid mixture transitions could not be recorded well below T_m. Here, due to the lack of extension on the low temperature side of our DSC experiment, we use the cooling scans to measure the main

transition temperature. Scans were generated with MCDSC-run software and analysed with NanoAnalyze (TA instruments). The main phase transition temperature was determined as the intersection of the base line and the main peak slope, as schematized in Fig. S6a.

Molecular Dynamics simulations:
A series of all-atom and coarse-grain molecular dynamics (MD) simulations have been used to study the effects of oxidized species of POPC on the structural and interfacial properties of model lipid bilayers. In doing so, we have modelled two oxidized species of POPC, one where the lipid is oxidized in the 9th carbon of the unsaturated tail (oxPOPC9) and the other when the 10th carbon of the unsaturated tail is oxidized (oxPOPC10), as shown in Fig. S15. When modelling oxidized lipids, we represent half of the oxidized lipids as oxPOPC9 and half as oxPOPC10. All molecular dynamics simulations were carried out using the GROMACS simulation engine.[10] Further details are given in the main text.

Analysis: The various analysis of the molecular dynamics simulations were carried out with the LiPyphilic Python package[11] and others scripts developed in-house which utilise MDAnalysis.[12,13]

A 2D Voronoi tessellation of atomic positions in each leaflet was performed to determine the APL of each component of the bilayer, using the C21 C2 and C31 atoms as seeds for the PC lipids (atoms are given by CHARMM atom names). We calculate the mean area per lipid (APL) of each species at each frame and calculate the standard deviation over time. The lipid order parameter is a measure of the conformational flexibility of acyl chains in a bilayer, and is given by:

$$S_{cd} = \frac{1}{2} \langle 3\,cos^2(\theta) - 1 \rangle \qquad \text{(s10)}$$

where θ is the angle between the bilayer normal and the carbon-hydrogen vector of a carbon atom in an acyl tail, and the average is taken over time and over all molecules of a given species within the membrane. The S_{CD} was calculated for each lipid species as a function of carbon atom position along an acyl chain. Smaller values of S_{CD} indicate a more disordered acyl chain.

In order to determine whether an acyl tail was snorkelling towards the water/bilayer interface, we first constructed a lateral 6 x 6 grid of the membrane, then found the difference in z from the terminal carbon atom to the carbon atom furthest from the interface. If this difference was greater than 5Å then we consider the tail to be snorkelling.

We calculated the lateral mean squared displacement (MSD) of each species using the C2 and O3 atoms of the PC lipids and ergosterol, respectively, over the final 500 ns of each simulation. Before calculating the MSD of each lipid, we first removed the lateral centre of mass motion of the bilayer. We then calculated the lateral diffusion coefficient (D) for each lipid using the Einstein relation:

$$D = \frac{1}{2d} \lim_{t\to\infty} \frac{\langle (r_t - r_{t0})^2 \rangle}{t} \qquad \text{(s11)}$$

where *d* is the system dimension, *r* is the coordinate of the C2 atom at a given time, *t*, from a time origin, *t0*. We then calculated the average and standard error of *D*. The MSD was calculated using the Fast Correlation Algorithm, implemented in tidynamics.[14] Finally the fractional lipid enrichment of species *i* around molecules *j*, E_{ij}, was measured using the associated tools within LiPyphilic.

2. Supplementary Figures

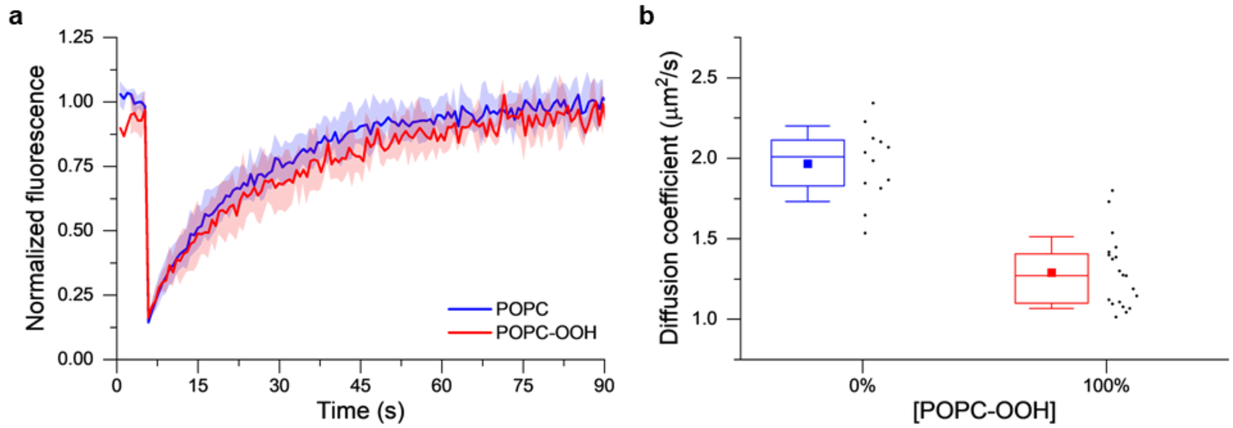

Fig. S1: FRAP measurements of lipid diffusion in POPC and POPC-OOH GUVs. (a) FRAP traces showing the mean and S.D. of example recovery curves (n=4). (b) Estimated diffusion coefficient of NBD-PE lipids in POPC and POPC-OOH bilayers. Box plots display the 25-75% range, error bars represent ±S.D., median is shown by a horizontal line and mean by a dot of N≥10 GUVs from n=3 independent repeats.

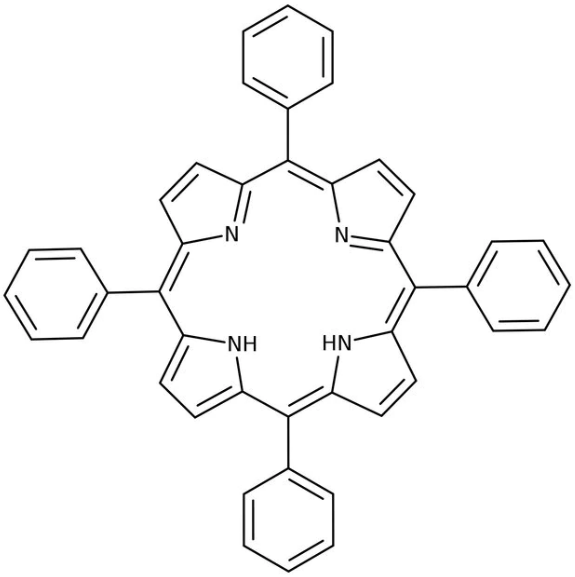

Fig. S2: Molecular structure of the photosensitizer tetraphenylporphyrin (TTP) used for *in situ* photooxidation experiments (420 nm TPP irradiation)

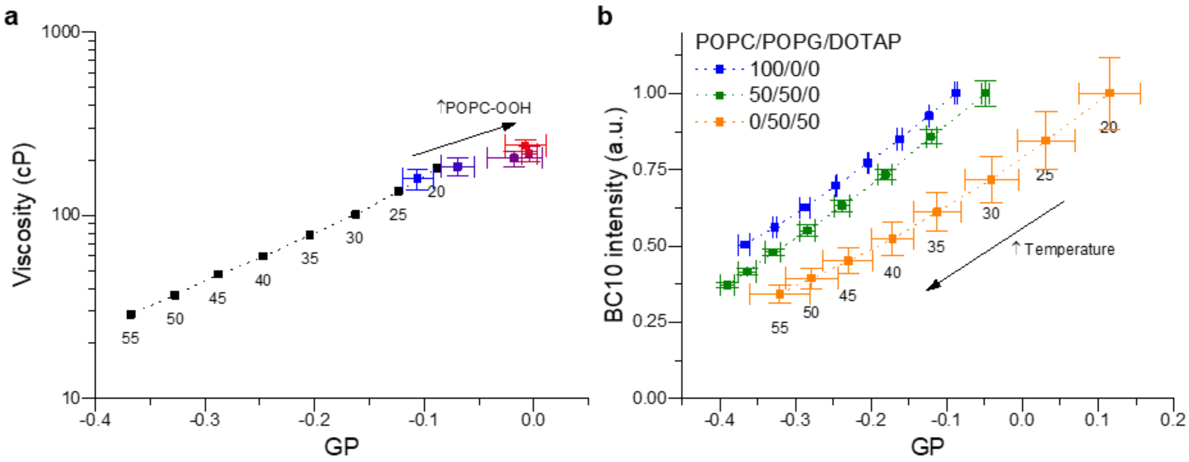

Fig. S3: The relationship between the BC10 rotor readout and Laurdan's GP. (a) The correlation between GP and microviscosity is similar in pure POPC bilayers at different temperatures (black trace and dots) and in POPC liposomes containing increasing amounts of POPC-OOH (coloured data points). This suggests that the interaction between -OOH and BODIPY rotors have a negligible influence on the readout of the rotor. (b) The relationship between the membrane polarity (as measured by Laurdan's GP) and the BC10 fluorescence intensity (proportional to the membrane viscosity), measured at a range of temperatures for liposomes containing charged lipids, 1-palmitoyl-2-oleoyl-sn-glycero-3-phospho-(1'-rac-glycerol) (sodium salt) (POPG) and 1,2-dioleoyl-3-trimethylammonium-propane (chloride salt) (DOTAP). The similarity between the three responses suggests that the BC10 sensitivity is not significantly affected by changes in membrane charge. Data shown as mean±S.D.

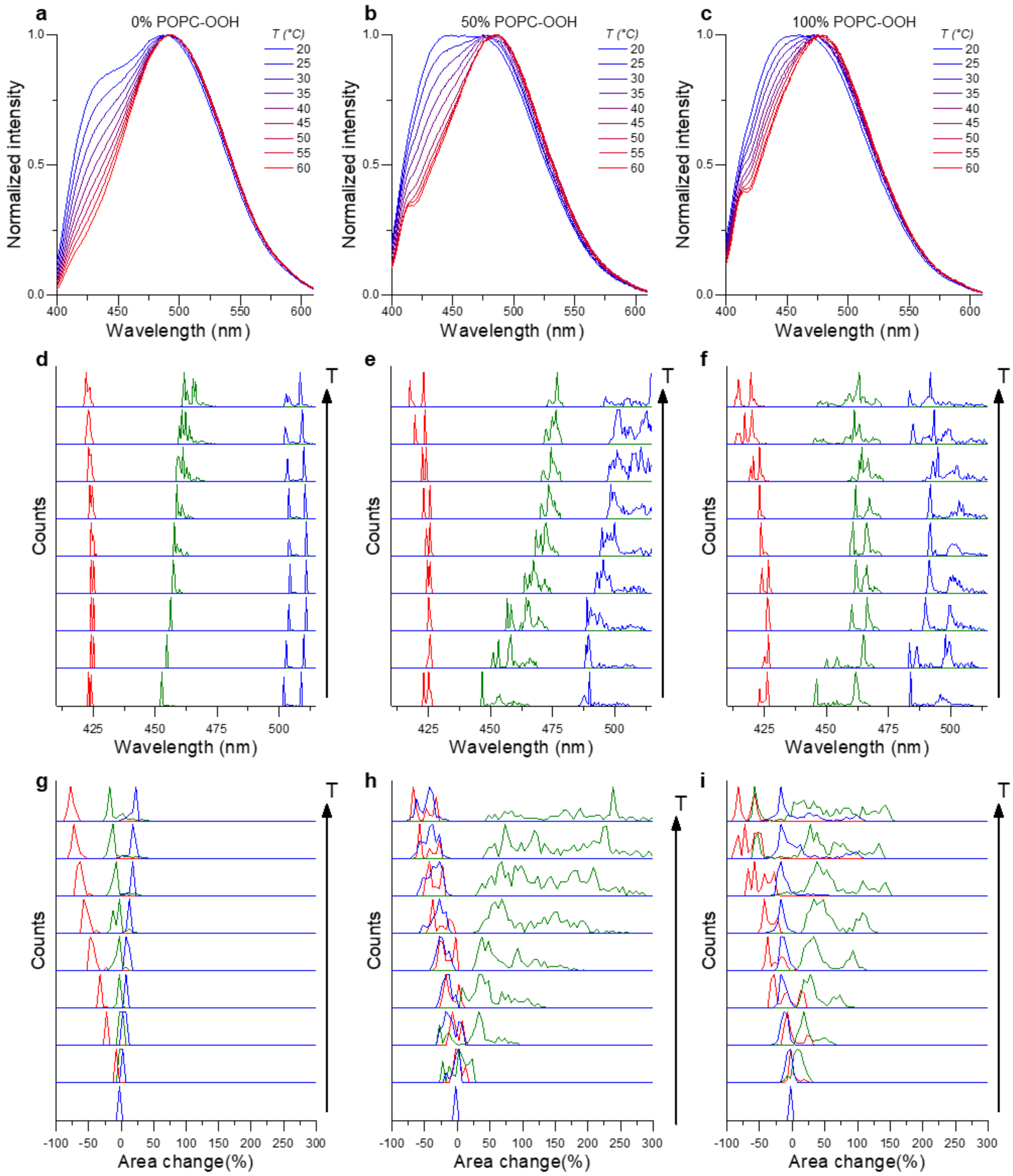

Fig. S4: The effect of temperature and POPC-OOH content (0, 50 and 100% of the oxidized lipid added to POPC) on Laurdan emission spectra (a-c) and its decomposition into apolar (red), polar aprotic (green) and polar protic (blue) components, performed according to the modified procedure[4] described in the Methods section. 100 starting conditions were sampled, and it is clear to see that irrespective of the starting conditions used, a shift in the component's wavelength (d-f) and relative contribution (g-i) at increasing temperature can be seen. This data confirms the increase in GP is compatible to -OOH looping towards the bilayer's surface.

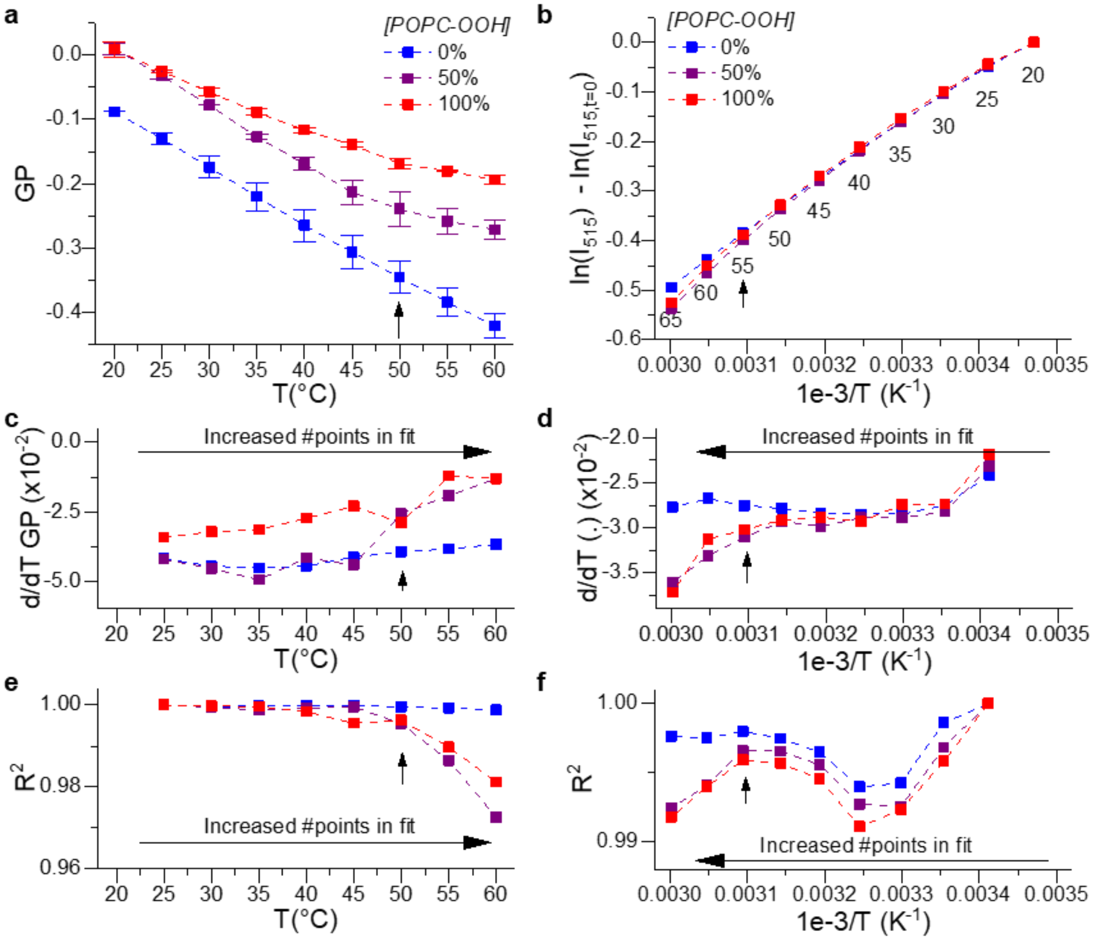

Fig. S5: Investigating the effect of temperature on POPC-OOH containing LUVs. (a) Change in Laurdan's GP; the change in the slope with increasing temperature is evident for POPC-OOH-containing membranes. (b) Change in **BC10** fluorescence intensity (which is proportional to viscosity); for a liquid following Andrade's relationship ($\mu = Ae^{\frac{B}{T}}$), the trace is expected to be linear. However, in the case of POPC-OOH-containing membranes the change in the slope was observed. The points corresponding to a change in the slope are marked with arrows. It could be seen that both methods record deviations from linearity in the slopes at the same temperature: ~50°C for 50% oxidized lipid and ~55°C for 100% oxidized lipid. Further evidence for the change in slope is highlighting in (c,d) the plots showing the first derivative (defined as $\frac{y(i)-y(i-1)}{x(i)-x(i-1)}, i \geq 2$) and in (e,f) where the goodness-of-fit parameter R^2 is shown for a linear fit to all GP or fluorescence intensity data between 25°C and the temperature of the data point. If there was no change in slope the addition of additional points (at higher temperatures) should have a minimal effect on the error metric and thus the error would not significantly change – as we see when evaluating pure POPC membranes. On the contrary, if a change of slope is present, the addition of fitting points in these regions will decrease the fitting quality in regions with a different trend, and this will be reflected by a lower goodness of fit, as we indeed see above ~50-55°C for POPC-OOH containing membranes. Data shown as mean±S.D (n=2 independent repeats).

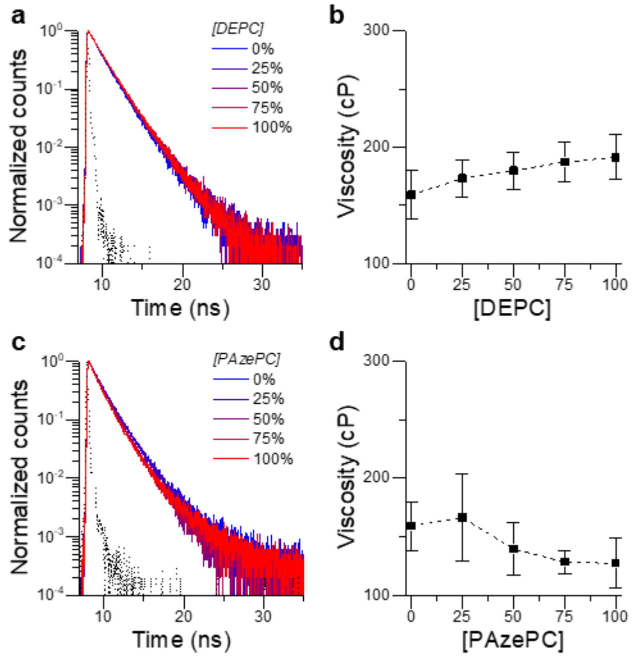

Fig. S6: Time resolved fluorescence decay traces of **BC10** and the corresponding calculated lipid membrane viscosity recorded for POPC /*trans* lipid DEPC mixtures (a,b) and POPC/truncated lipid PAzePC mixtures (c,d). A small increase in viscosity in seen upon an increase in DEPC fraction, which is, however, significantly smaller than the increase seen upon POPC-OOH addition (Fig. 2, main text). As expected, a decrease in viscosity is seen upon an increase in PAzePC fraction. Data shown as mean±S.D (n=3)

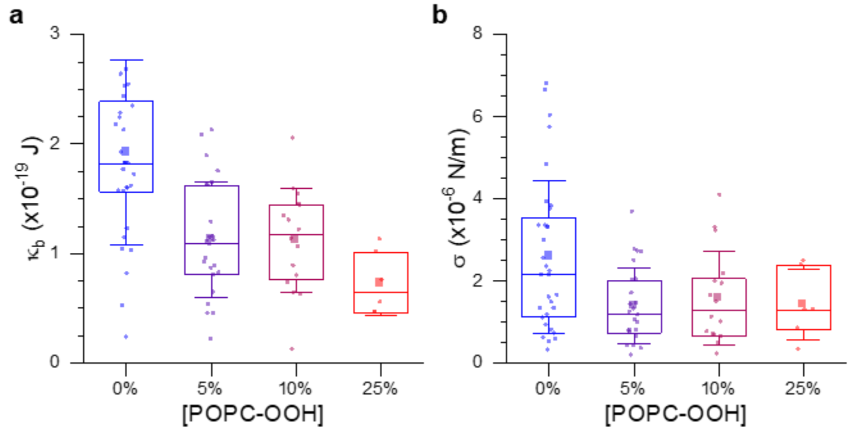

Fig. S7: Flickering spectroscopy analysis of POPC-OOH containing GUVs using a two-parameter fit. Bending rigidity decreases with an increased fraction of lipid peroxide (a), while no significant changes in membrane tension could be detected (b). Box plots display the 25-75% range, error bars represent ±S.D., median is shown by a horizontal line and mean by a dot. N≥5 from n=2 independent experiments.

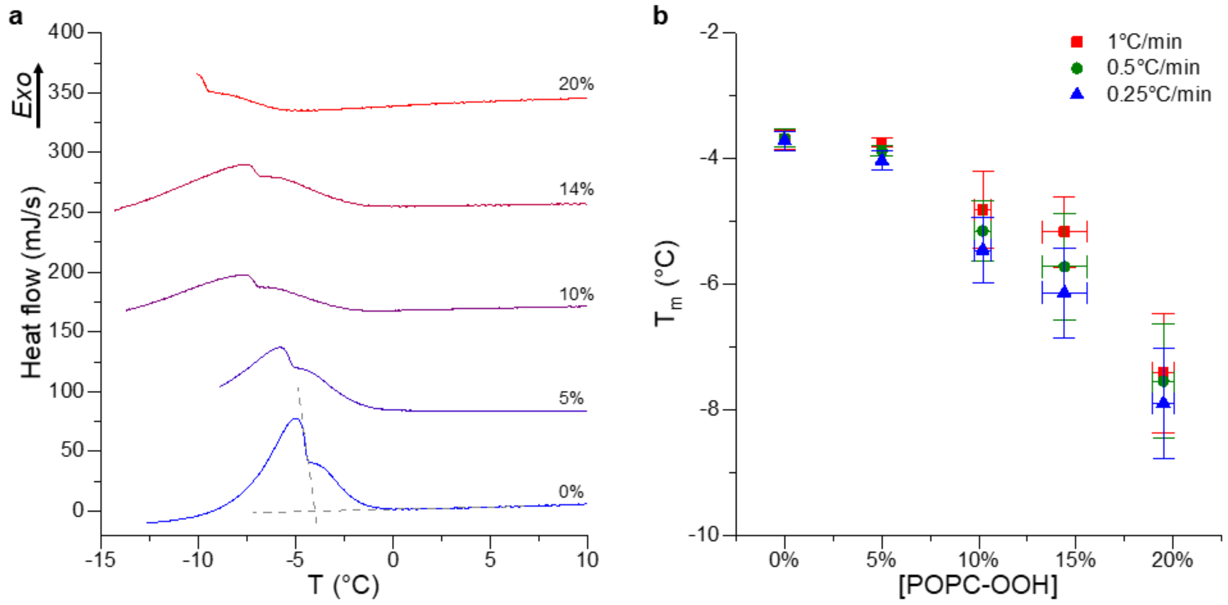

Fig. S8: a) DSC cooling scans of POPC:POPC-OOH at increasing fractions of lipid peroxide. Dashed lines show an example of the intersection between the baseline and the main peak slope. Scans were performed at 0.25 ºC/min. b) Effect of peroxidation on transition temperature T_m measured at different C/H rates. The decrease in T_m indicates the disrupting effect of POPC-OOH on POPC packing. Data shown as mean±S.D (n=3 independent repeats).

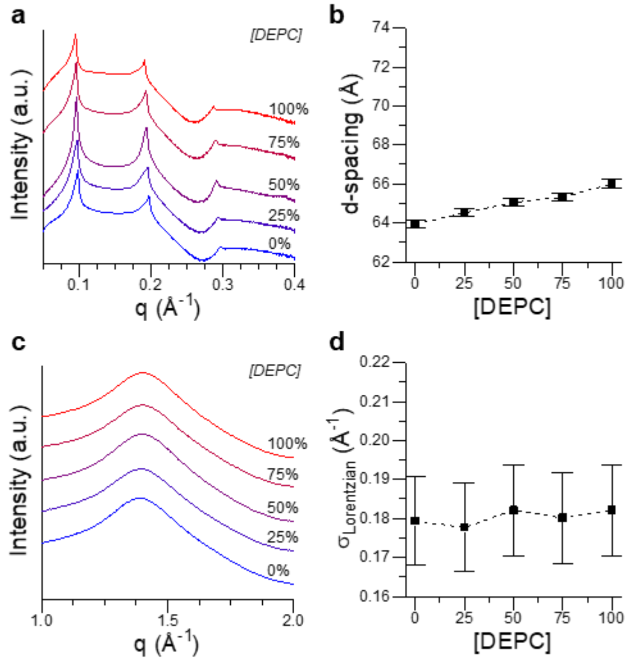

Fig. S9: X-ray scattering intensity profiles for POPC/DEPC lipid stacks recorded at 70% hydration. (a, b) SAXS profiles and interlamellar spacing calculated from the 1st Bragg peak; (c, d) WAXS traces and the width of the fitted Lorentzian component. This data indicates the trans-nature of POPC-OOH does not account for the observed increase in interlamellar spacing and in membrane heterogeneity. Data shown as mean±S.D (n=3) S.D. in DEPC containing membranes was estimated according to the S.D. of pure POPC samples, see ESI – Additional Experimental methods for details.

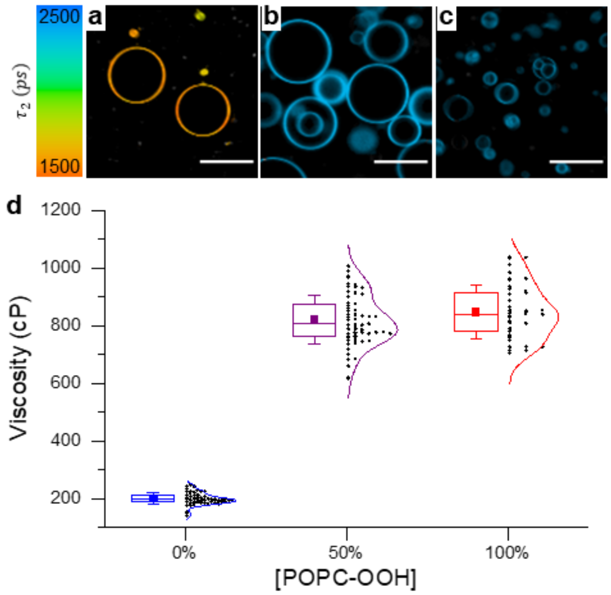

Fig. S10: (a-c) Example FLIM images of **BC10** stained POPC/POPC-OOH GUVs recorded for increasing POPC-OOH fractions; (d) Calculated distribution of membrane viscosities (each point corresponds to a separate GUV). Scale bar: 30µm. Initially, we mixed **BC10** with the POPC/POPC-OOH lipids in chloroform and prepared dry films of the mixture, before forming GUVs *via* electroformation. However, we observed unexpectedly high **BC10** lifetimes for POPC-OOH containing vesicles prepared this way, which corresponded to extremely high viscosity values, in clear disagreement with previous data.[2] This artefact was not observed when the membrane was stained post-electroformation with well characterized water-soluble molecular rotor **BC6++** (Fig. 5, main text). Box plots display the 25-75% range, error bars represent ±S.D., median is shown by a horizontal line and mean by a dot. N≥20 from n=2 independent experiments.

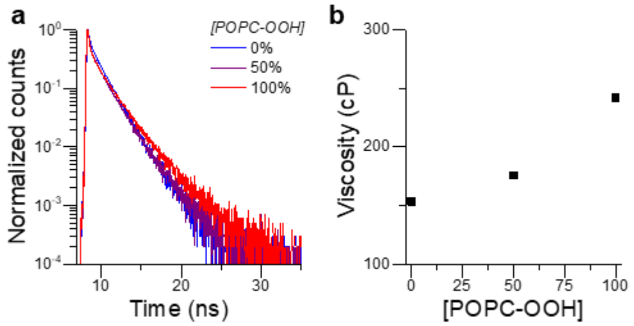

Fig. S11: Decay traces (a) and calculated viscosity (b) of **BC6++** stained POPC LUVs at increasing POPC-OOH fraction. The obtained values are comparable to those obtained using **BC10**.

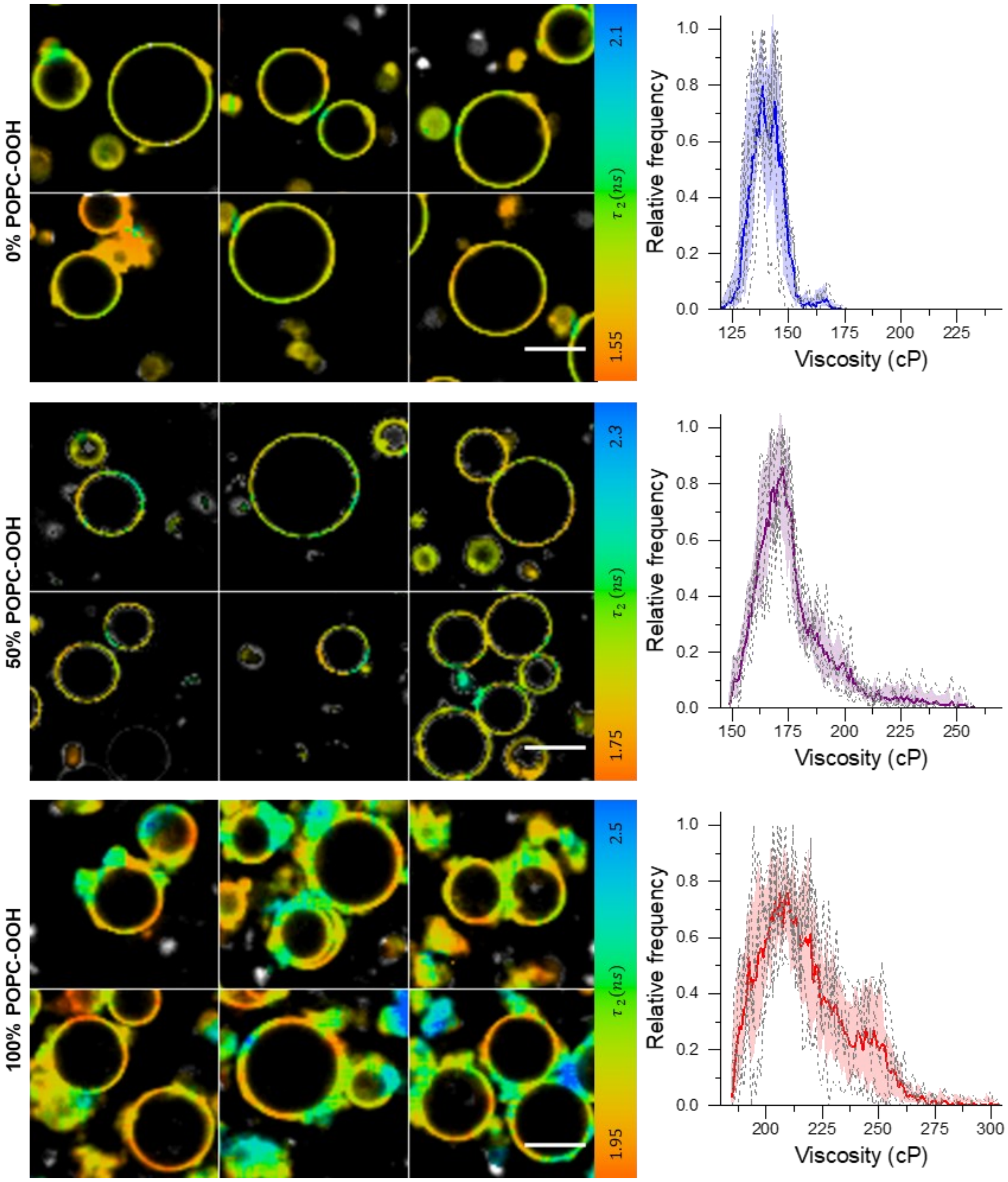

Fig. S12: POPC-OOH increases lateral heterogeneity in model GUVs. (a) Example of FLIM images. (b) Histograms representing the average of the pixel-wise lifetimes in each of the images displayed in (a). Scale bar: 20 µm.

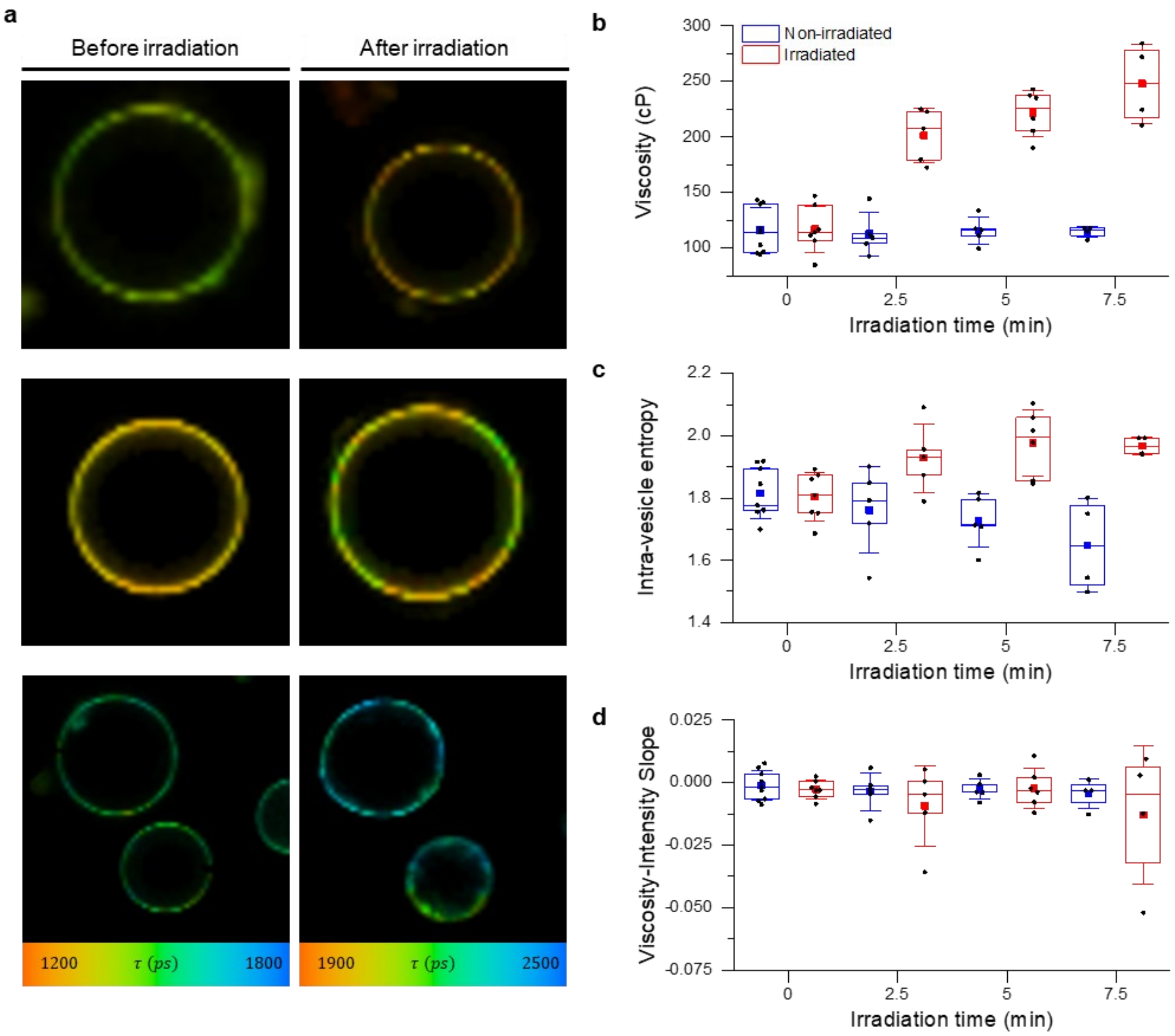

Fig. S13: FLIM gives evidence for the appearance of highly ordered lipid clusters in photooxidized POPC GUVs. (a) FLIM images of different GUVs stained with **BC10** and TPP before/after 900 nm 2-photon irradiation for up to 7.5 min. Nonhomogeneous colour (=lifetime) distribution across each irradiated GUV gives evidence for the domain formation. (b) GUV viscosity recorded at different irradiation times, the total of 5 different vesicles were analysed. (c) Quantification of the heterogeneity in membrane's viscosity. An increase in Shannon's entropy ($\log_{10}$ based) is observed for the irradiated GUVs, suggesting a considerably more heterogeneous viscosity distribution, compared to equivalent non irradiated GUVs, which is consistent with lipid clusters of different fluidity appearing upon photooxidation. (d) Verification that the polarization-related artefacts do not play role in the observed heterogeneity. We plotted the slope resulting from linear fitting of the viscosity vs intensity data for each analysed GUV. The slope remains close to zero for all conditions throughout the irradiation experiment, i.e. there is no correlation between the lifetime and intensity in the images. Box plots display the 25-75% range, error bars represent ±S.D., median is shown by a horizontal line and mean by a dot. (N≥3 GUVs in n=3 independent repeats).

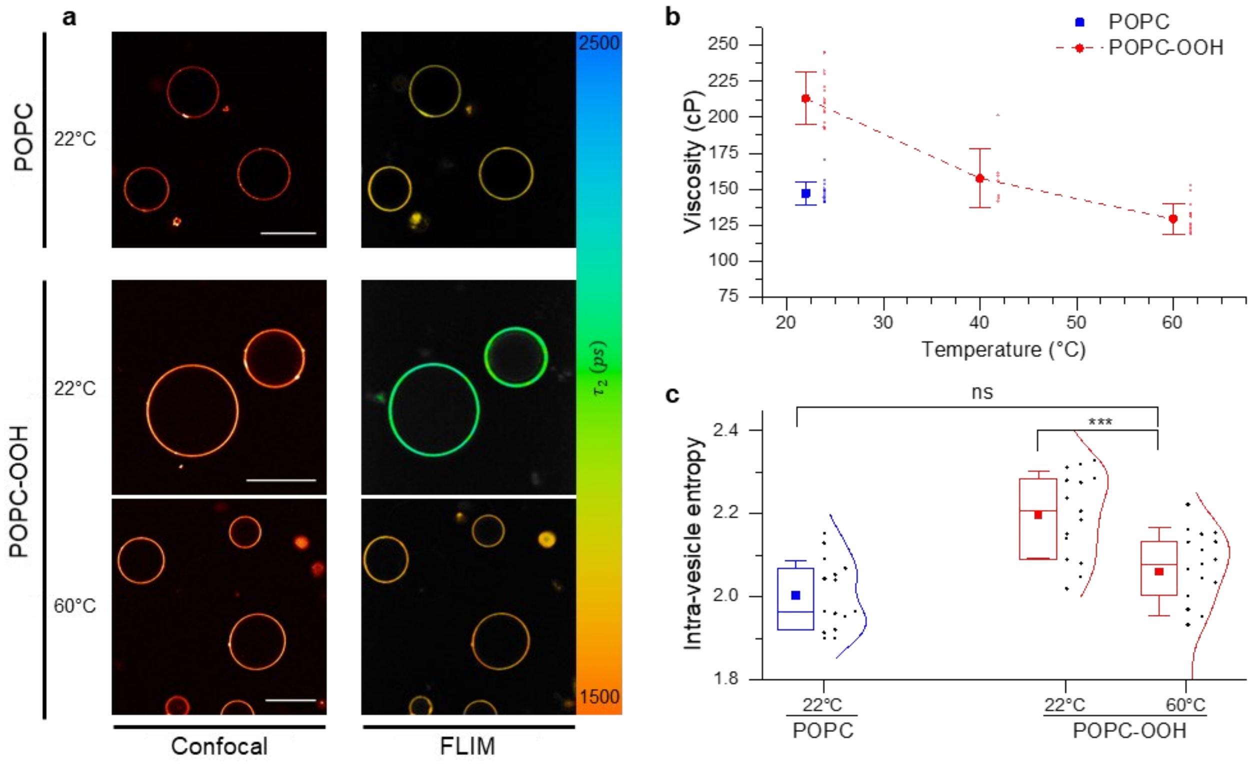

Fig. S14: Temperature effect on POPC-OOH GUVs viscosity and lateral heterogeneity. a) Confocal and FLIM images of POPC and POPC-OOH at 22°C, and of POPC-OOH at 60°C. At low temperature, membrane viscosity corresponding to POPC-OOH GUVs appears to be heterogeneous, while this effect is not seen for POPC (at 22°C). Additionally, POPC-OOH vesicles lose heterogeneity at higher temperature (60°C) when no lipid clustering is expected, according to our earlier data, Fig. S2. Scale bar: 20µm b) Change of POPC-OOH viscosity with temperature. The change in slope suggests the existence of POPC-OOH rich lipid clusters. Data shown as mean±S.D (N≥10 GUVs in n=2 independent repeats). c) Shannon's entropy (log_{10} based) is used to quantify the intra-vesicle viscosity heterogeneity. At higher temperatures, the increased bilayer homogeneity of POPC-OOH membranes leads to a decrease in entropy, which becomes comparable to that of POPC vesicles. Box plots display the 25-75% range, error bars represent ±S.D., median is shown by a horizontal line and mean by a dot. (N≥10 GUVs in n=2 independent repeats).

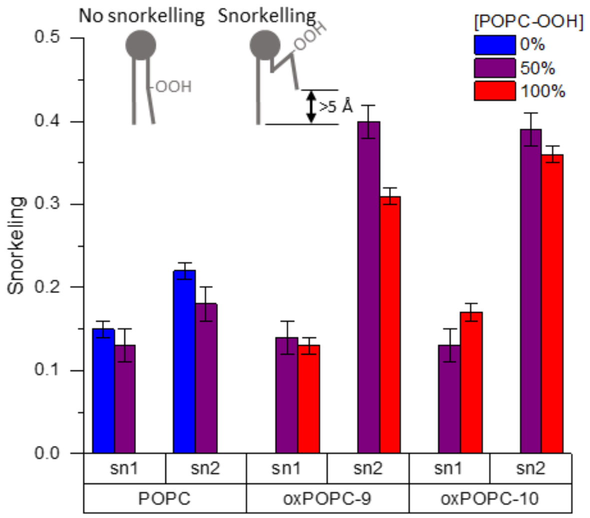

Fig. S15: Snorkelling probability of the lipid chains calculated from the MD simulations for mixtures of POPC with increasing concentration of POPC-OOH. SN2 chain of oxidized lipids show increased likelihood to snorkel. Snorkelling is likely to increase hydrocarbon chain density at the surface of the bilayer, which can serve as a mechanism for domain formation. Data shown as mean±S.D (N≥10 GUVs in n=2 simulations)

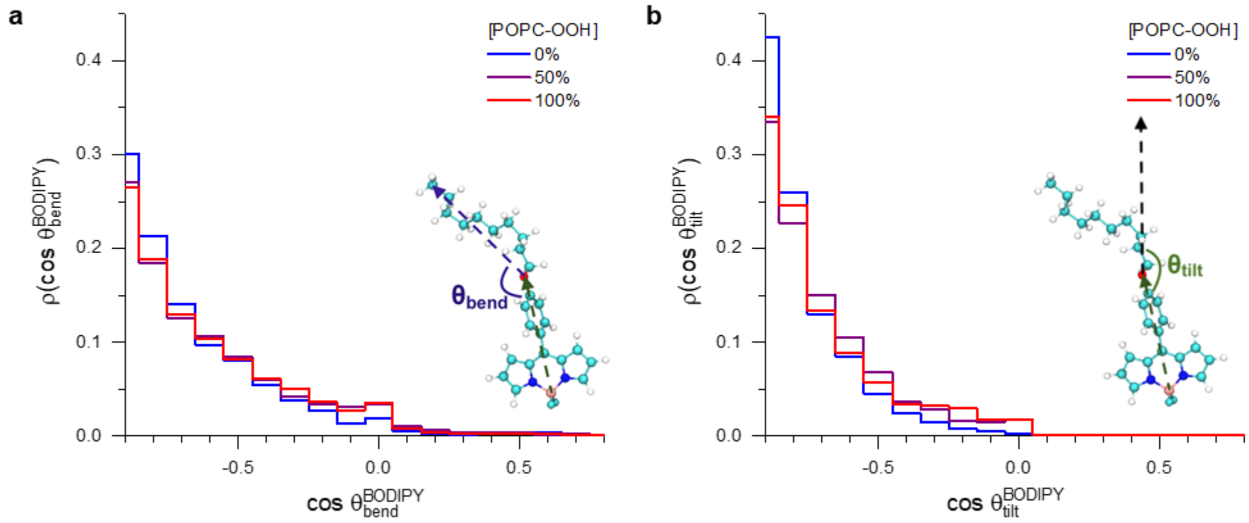

Fig. S16: Bending (a) and tilt (b) angle distribution of **BC10** embedded in lipid bilayers with increased POPC-OOH concentration. This data indicates that no positional changes of **BC10** is observed in the oxidized compared to pure POPC bilayer, confirming that the observed lifetime (thus viscosity) changes relate to a changing viscosity rather than the re-localization of the sensor.

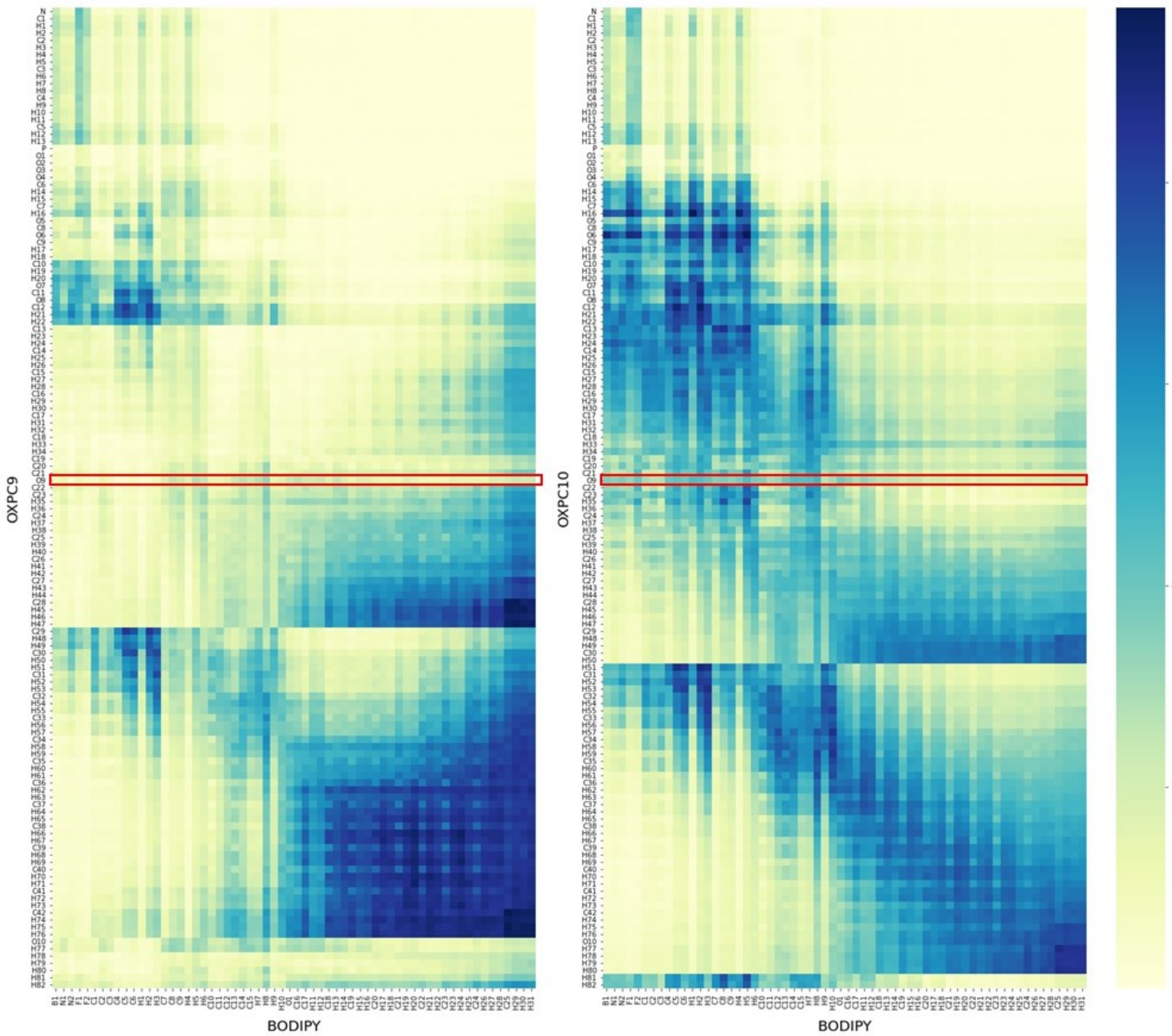

Fig. S17: Interaction map between BC10 and POPC-OOH. The highlighted row, corresponding to the oxygen in the peroxide, shows a lack of significant bonding between the dye and the oxidized lipid.

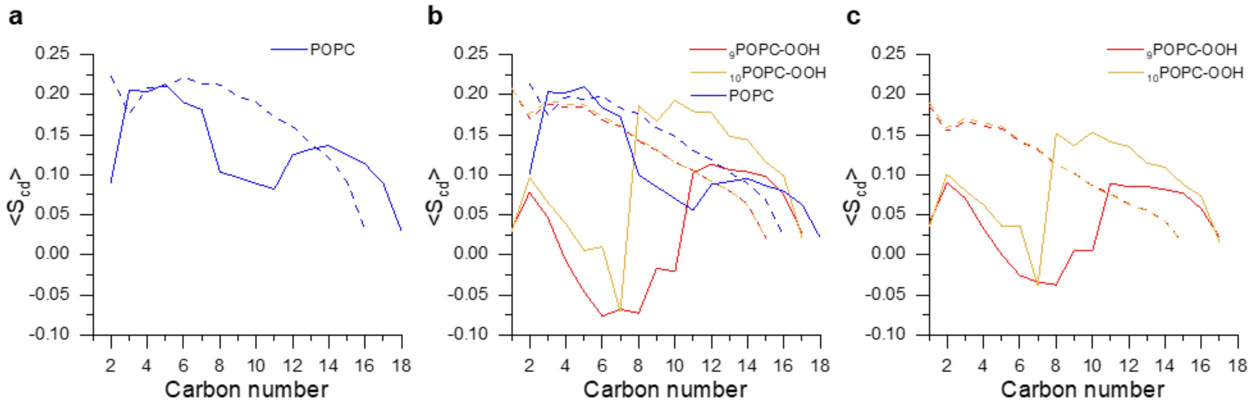

Fig. S18: Average lipid order parameter Scd for the membrane compositions with increasing POPC-OOH concentration. Continuous traces correspond to unsaturated sn2 chains, dashed traces to saturated sn1 chains. a) 0% POPC-OOH; b) 50% POPC-OOH; c) 100% POPC-OOH. The snorkelling of the sn2 hydroperoxidized chains is reflected by the increase in ordering at the carbon positions 8 and 11.

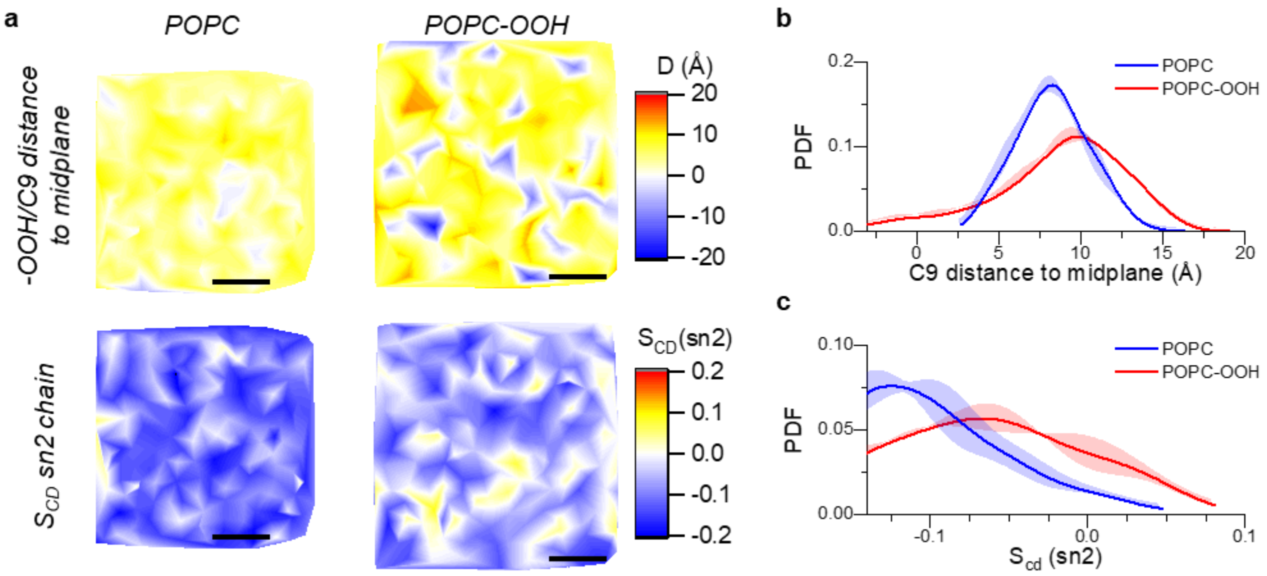

Fig. S19: Lipid clustering in POPC-OOH membranes. a) 2D maps showing the distance between the double bond/-OOH group to the membrane's midplane (top) and the order parameter (S_{cd} (sn2)) of the unsaturated chain for POPC and POPC-OOH membranes. Surface heterogeneity, which is indicative of lipid domains, is evident in the presence of oxidized lipids. Scalebar: 30nm. b) Probability distribution of the C9 distance to midplane and c) probability distribution of the S_{cd} (sn2) order parameter for POPC and POPC-OOH bilayers.

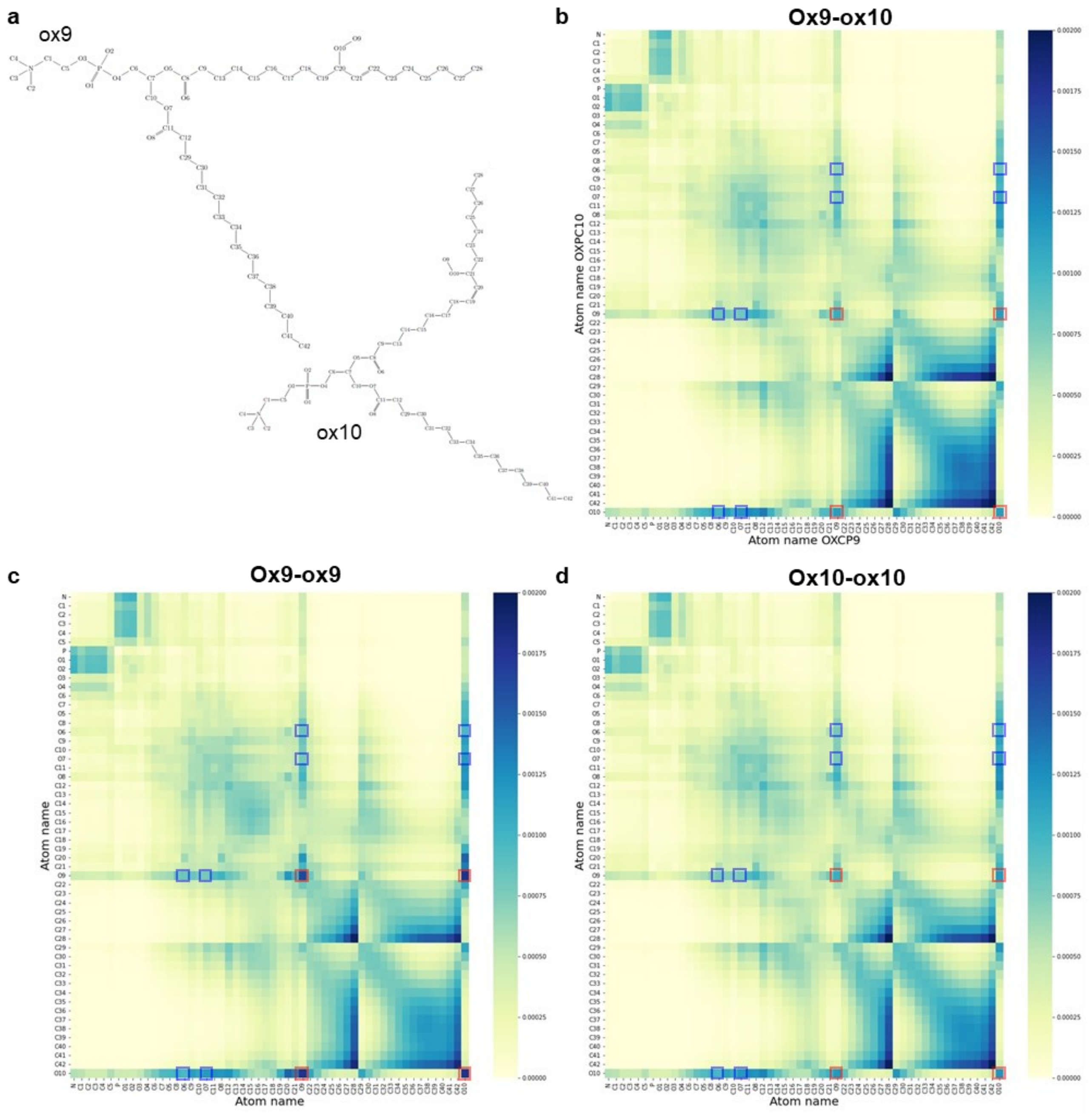

Fig. S20: Contact maps between the two POPC-OOH species. Red squares mark the interactions between oxygens in the oxidized tails (O9, O10), while blue squares point to the contact between oxygens in the oxidized tails with those in the ester groups (O6, O7). Darker pixels in the heatmap represent a larger extent of hydrogen bonding between the indicated species. a) Molecular structures of the two POPC-OOH variations employed throughout the simulations, ox9 and ox10. Contact maps are shown between b) ox9-ox10; c) ox9-ox9 and d) ox10-ox10. These contacts indicate the H-bond mediated interaction between the -OH in with the oxidized tails and ester groups of neighbouring lipids, a potential mechanism for clustering of oxidized lipid species.

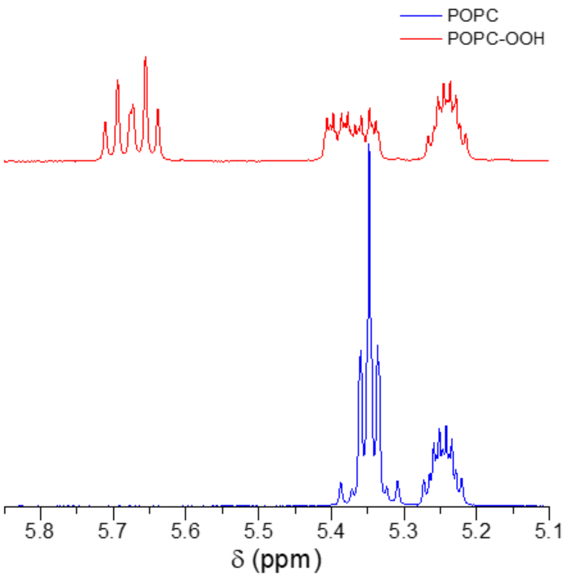

Fig. S21 Part of the 1H-NMR spectrum of pure POPC and POPC-OOH, used to identify the hydroperoxidation rate of the chains (400 MHz, CD_3OD).

3. Supplementary References:

1. Soumpasis, D. M. Theoretical analysis of fluorescence photobleaching recovery experiments. *Biophys. J.* **41**, 95–97 (1983).

2. Dent, M. R. *et al.* Imaging phase separation in model lipid membranes through the use of BODIPY based molecular rotors. *Phys. Chem. Chem. Phys.* **17**, 18393–18402 (2015).

3. Saffman, P. G. & Delbruck, M. Brownian motion in biological membranes. *Proc. Natl. Acad. Sci.* **72**, 3111–3113 (1975).

4. Watanabe, N. *et al.* Solvatochromic Modeling of Laurdan for Multiple Polarity Analysis of Dihydrosphingomyelin Bilayer. *Biophys. J.* **116**, 874–883 (2019).

5. Camesasca, M., Kaufman, M. & Manas-Zloczower, I. Quantifying Fluid Mixing with the Shannon Entropy. *Macromol. Theory Simulations* **15**, 595–607 (2006).

6. Brandani, G. B. *et al.* Quantifying Disorder through Conditional Entropy: An Application to Fluid Mixing. *PLoS One* **8**, (2013).

7. Hosny, N. A. *et al.* Direct imaging of changes in aerosol particle viscosity upon hydration and chemical aging. *Chem. Sci.* **7**, 1357–1367 (2016).

8. López-Duarte, I., Vu, T. T., Izquierdo, M. A., Bull, J. A. & Kuimova, M. K. A molecular rotor for measuring viscosity in plasma membranes of live cells. *Chem. Commun.* **50**, 5282–5284 (2014).

9. Mills, T. T. *et al.* Order parameters and areas in fluid-phase oriented lipid membranes using wide angle x-ray scattering. *Biophys. J.* **95**, 669–681 (2008).

10. Van der Spoel, D. & Hess, B. GROMACS-the road ahead. *Wiley Interdiscip. Rev. Comput. Mol. Sci.* **1**, 710–715 (2011).

11. Smith, P. & Lorenz, C. D. LiPyphilic: A Python Toolkit for the Analysis of Lipid Membrane Simulations. *J. Chem. Theory Comput.* **17**, 5907–5919 (2021).

12. Michaud-Agrawal, N., Denning, E. J., Woolf, T. B. & Beckstein, O. MDAnalysis: A toolkit for the analysis of molecular dynamics simulations. *J. Comput. Chem.* **32**, 2319–2327 (2011).

13. Gowers, R. *et al.* MDAnalysis: A Python Package for the Rapid Analysis of Molecular Dynamics Simulations. *Proc. 15th Python Sci. Conf.* 98–105 (2016). doi:10.25080/majora-629e541a-00e

14. de Buyl, P. tidynamics: A tiny package to compute the dynamics of stochastic and molecular simulations. *J. Open Source Softw.* **3**, 877 (2018).
